# Enhancing Multi-Camera People Detection by Online Automatic Parametrization Using Detection Transfer and Self-Correlation Maximization †

**DOI:** 10.3390/s18124385

**Published:** 2018-12-11

**Authors:** Rafael Martín-Nieto, Álvaro García-Martín, José M. Martínez, Juan C. SanMiguel

**Affiliations:** Video Processing and Understanding Laboratory (VPULab), Universidad Autónoma de Madrid, 28049 Madrid, Spain; rafael.martinn@uam.es (R.M.-N.); josem.martinez@uam.es (J.M.M.); juancarlos.sanmiguel@uam.es (J.C.S.)

**Keywords:** self-correlation maximization, multi-camera, people detection, automatic parametrization

## Abstract

Finding optimal parametrizations for people detectors is a complicated task due to the large number of parameters and the high variability of application scenarios. In this paper, we propose a framework to adapt and improve any detector automatically in multi-camera scenarios where people are observed from various viewpoints. By accurately transferring detector results between camera viewpoints and by self-correlating these transferred results, the best configuration (in this paper, the detection threshold) for each detector-viewpoint pair is identified online without requiring any additional manually-labeled ground truth apart from the offline training of the detection model. Such a configuration consists of establishing the confidence detection threshold present in every people detector, which is a critical parameter affecting detection performance. The experimental results demonstrate that the proposed framework improves the performance of four different state-of-the-art detectors (DPM , ACF, faster R-CNN, and YOLO9000) whose Optimal Fixed Thresholds (OFTs) have been determined and fixed during training time using standard datasets.

## 1. Introduction

People detection is one of the main tasks of computer vision with applications in many areas such as video-surveillance or human-computer interaction. Such detection is difficult due to the variety of people appearance and pose, and its performance is also very dependent on the data used for training [1]. Classical people detection techniques can be divided into three stages [2]: firstly, a person model is designed that defines the characteristics that the detected objects must fulfill to be considered people; secondly, an object extraction process is performed, which will find the candidates to be classified; finally, the classification consists of the comparison of the objects detected in the sequence with the model generated in the first step. In this last step, a decision is made about the objects, and it is decided whether the objects are classified as persons or not. Depending on the application, the decision can be binary or a probability value of being a person.

The information provided by a single camera is limited, so in order to monitor a wide area or to obtain more information from the different viewpoints of a region of interest, it is necessary to use more than one camera. For this reason, the use of several cameras is a common way of developing applications [3,4], since it is also useful for solving occlusions in scenarios with a high density of people/objects and for 3D applications [5,6]. The use of a multi-camera environment in scenarios with possible occlusions usually improves the detection performance with respect to the use of the cameras independently. A method is proposed in [7] to perform detection and tracking of people in multi-camera environments where there are occlusions. This method is based on the methodology proposed in [8] and is based on using the information of each of the cameras from the scenario, merging it into a common plane (the ground plane) obtained by homographies. The individual information that is combined in the common plane is previously obtained by subtracting the background. Then, the object detections are performed in the common plane, and, afterwards the correspondence between cameras and objects is made. In this way, using cameras with different locations, the problem of occlusion is solved. The main limitation is that the individuals have to appear initially isolated. In [9], an improvement of the previous method [8] to eliminate false positives was proposed. Firstly, the algorithm that performs this process compares the views of all the cameras for each one of the detected objects, and then, it is able to avoid false detection by applying multiple view perspective geometry of people presence on the ground plane. It is also interesting to consider [10], where a method that uses a Kalman filter to obtain 3D information from the 2D information is presented.

Unlike the previous approaches, in this work, we propose to transfer the detections from one camera to another instead of just projecting all the detections to the common plane. In the state of the art, the information of the detections is usually projected in the ground plane at the point level (one point per detection) or at the mask level (masks are projected, and the intersections indicate the position of the detected person). The work presented in this paper considers the common plane to obtain the different camera views’ information, and it allows transferring (and afterwards, correlating) people detections from each camera to the other ones.

By employing multiple cameras, the available viewpoints provide additional information that may allow overcoming the limitations of detectors applied to single camera views. However, determining the confidence of the information generated for each viewpoint and, therefore, the automatic parametrization remains a challenging problem. Traditionally, optimal parameters are determined and fixed previously during training time [11,12,13,14]. A method is proposed in [15] to adapt people detectors automatically during runtime classification. The authors propose a mono-camera approach based on the correlation and combination of six detectors in order to choose frame by frame each detector threshold properly.

In this paper, we also propose a method to adapt the configuration of people detectors automatically during runtime detection. Unlike generic approaches fixing confidence thresholds or approaches restricted to single camera limitations, this method adapts the detector’s threshold for each frame and camera. We consider generic threshold-based detectors, trained on standard datasets, making this proposal applicable to most state-of-the-art people detectors.

This paper is organized as follows: Section 2 overviews the proposed approach and main contributions, whereas Section 3 and Section 4 describe the detection transfer between cameras and the correlation framework. Section 5 presents the experiments. Finally, Section 6 concludes this paper.

## 2. Framework Overview

**Figure 1:**
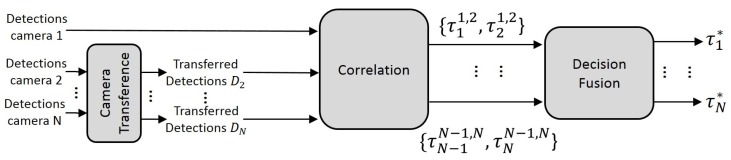
Framework overview.

In [15], a method is proposed to select, based on the correlation of multiple detectors, the working threshold in runtime. Note that the work in [15] is based on the correlation of pairs of detectors and not pairs of cameras, and it does not consider the information transfer between cameras. The new proposed approach uses the correlation stage just as part of the whole framework (see Figure 1). The proposed framework is able to transfer detections to a different point of view, to combine multiple cameras automatically and to select the working threshold of each one of them automatically in runtime. Firstly, the system is able to extract the information of each camera independently, i.e., each camera detects independently. Afterwards, a generic transfer algorithm is proposed in order to establish a common point of view and to concentrate the information of every camera. Then, the corresponding correlation between pairs of cameras is performed in the common field of view of all cameras. Finally, the correlation stage is able to determine automatically the best threshold for each camera simultaneously. Figure 1 shows the complete framework. The different parts of the framework, which are described in more detail in their corresponding sections, are the following:

1. The frame-by-frame detections of all cameras are extracted, transferred, and homogenized (the position and volume of the transferred detections between cameras must be corrected) to the desired viewpoint ${\left\{D_{n}\right\}}_{n=1}^{N}$. In this way, the object information is not reduced to a simple coordinate, allowing transferring more information (volume, height, aspect ratio) and processing the information for each camera viewpoint.
2. The homogenized detections from the previous stage are correlated frame by frame, and an optimal decision threshold is selected for each camera and frame. The correlations are computed for each pair of transferred detection results ($D_{n}$ and $D_{m}$), which determine an optimal pair of thresholds for each pair of cameras ($\tau_{n}^{n,m}$ and $\tau_{m}^{n,m}$, respectively). Finally, the pair-wise selected thresholds are combined by weighted voting to obtain the best adapted threshold for each individual camera ($\tau_{1}^{*}$, ..., $\tau_{N}^{*}$).

## 3. Detection Transfer between Cameras

**Figure 2:**
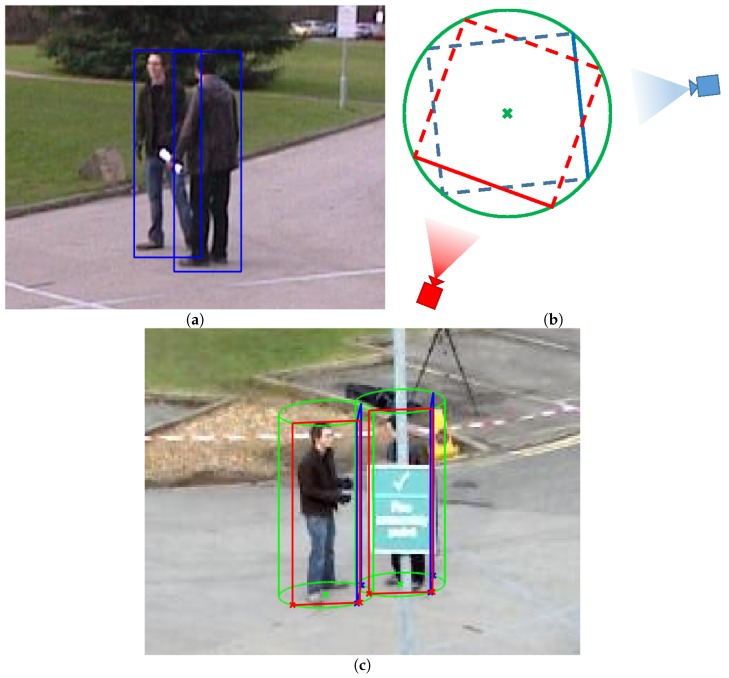
Overview of the proposed technique: (**a**) shows two detection bounding box examples; (**b**) schematizes the geometric process; and (**c**) contains a representation of the resulting cylinders (green), the original bounding box (blue, very tilted due to the angle between the cameras’ viewpoints), and the resulting bounding boxes (red). (**a**,**c**) are cropped versions for visualization purposes.

A cylinder is considered to approximate the location and volume of a person in order to transfer the position of the detection bounding boxes from one camera to another, maintaining the volume that a person occupies, instead of using only the projected plane generated from the detected bounding box. The consideration of the representation of people as cylinders has been used previously in the state of the art [16], but as a method for people counting (estimation) from a single camera perspective. The objective of the developed technique is to transfer the bounding boxes of the detections from one camera to the viewpoint of another camera. As the projections on the common plane of the detected bounding boxes do not correspond spatially with the position and volume of the detected object, the transfer between cameras must be corrected. Figure 2a shows two bounding boxes that will be transferred. In Figure 2b, each projected bounding box base is represented with the continuous blue line, and the cylinder base is represented with a (green) circle. The continuous red line corresponds to the projection of the transferred bounding box base and belongs to the rotated (red) square. An example of the resulting cylinders is shown in Figure 2c. Here, we describe the method applied to each bounding box detected by the camera whose information is transferred.

1. Firstly, the base (bottom) segment of the detection bounding box is projected to the common plane, the ground floor plane in our scenario. This plane can be obtained using homographic techniques, or from the intrinsic and extrinsic parameters of the cameras. We use the base segment as it is in the common plane (we assume that every person is over the ground and therefore also the base segment of their corresponding bounding box), which allows accurately transferring it. Figure 2a shows two bounding boxes that will be transferred.
2. Using the projected segment in the common plane, a circumference is defined so that the projected segment forms one of the sides of a square inscribed therein. In Figure 2b, the projected segment is represented with the continuous blue line, the square is represented with the discontinuous blue line, and the circumference is represented with a (green) circle.
3. To define the bounding box base segment that will be transferred to the other camera, the inscribed square (blue) is rotated (represented with the discontinuous red line in Figure 2b) with an angle such that the closest side is perpendicular to the line connecting the new camera with the center of the circumference (green cross in Figure 2b). This improves the direct transfer results, i.e., without geometry and volume considerations. This side (continuous red line) corresponds to the projection of the transferred bounding box base segment.
4. The height of the cylinder is estimated assuming a fixed aspect ratio, taking into account the object original height and the cameras’ distances to the object.
5. Finally, this generated cylinder is transferred to the point of view of the new camera, again using a homography (inverse matrix) or from the intrinsic and extrinsic parameters of the new camera. An example of the resulting cylinders and transferred bounding boxes is shown in Figure 2c.

## 4. Correlation Stage

We apply a method to improve the detection performance at runtime by adapting the detector configuration (see Figure 1). This proposal is based on the maximization of mutual information strategy where classifiers are combined assuming that their errors are complementary [15]. In our case, the detection model, executed in the different cameras, has been trained using the same content set. The incorrect detections will be different for each camera, so the correlation will reinforce the correct detections common to all cameras and penalize the isolated errors of each camera.

We start from a set of *N* camera frames. Each detector obtains a confidence map in every camera, $M_{n}$, representing the likelihood of people presence at each spatial location in the frame. Then, detection candidates are obtained by thresholding this map. Each detection (i.e., bounding box) is described by its position (x,y) and dimensions (w,h). The set of detections are transferred to the camera under analysis (i.e., the desired viewpoint) ${\left\{D_{n}\right\}}_{n=1}^{N}$. The transferred camera detections are compared to obtain a set of pairwise correlation scores. Firstly, the decision space of each camera output is explored by applying multiple thresholds. Then, these multiple outputs are correlated for each pair of camera detections ($D_{n}$ and $D_{m}$) to obtain a correlation map, which measures the output similarity. Finally, the configuration with the highest similarity allows selecting the best detection threshold for each camera output ($\tau_{n}^{n,m}$ and $\tau_{m}^{n,m}$, respectively). Up to this point, we have a hypothesis obtained for each compared pair of detections ($D_{n}$ and $D_{m}$), which are combined to obtain a final configuration for each camera threshold ($\tau_{1}^{*}$, ..., $\tau_{N}^{*}$). Such a hypothesis combination is performed as a traditional mixture of experts via weighted voting in the decision fusion stage as follows:

(1) $$\tau_{n}^{*}=\sum_{m=1}^{N}\omega^{n,m}\cdot \tau_{n}^{n,m}\;\left(n\neq m\right).$$

where $\omega^{n,m}\in \left[0,1\right]$ is the weight for the hypothesis $\tau_{n}^{n,m}$ achieved by comparing $D_{n}$ and $D_{m}$ and $\sum_{m=1}^{N}\omega^{n,m}=1\;\left(n\neq m\right)$. Currently in this work, we assume no prior knowledge about cameras’ performance, so we consider equal weighting $\omega^{n,m}=\frac{1}{N-1}$.

**Figure 3:**
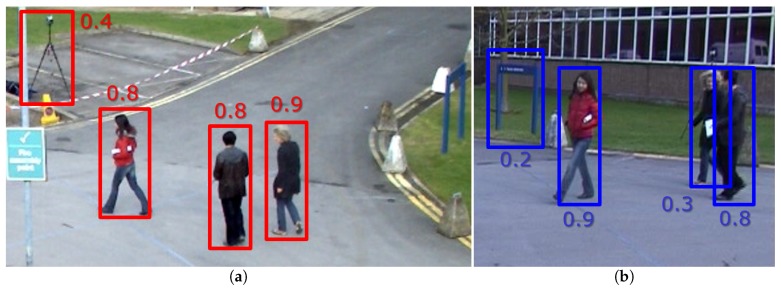

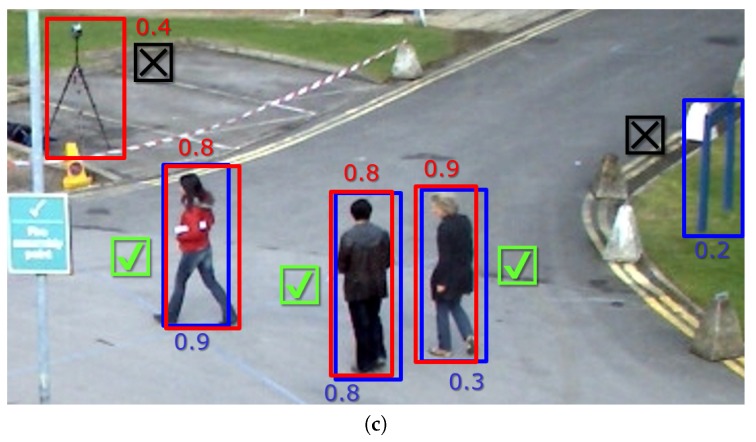
Visual example of the correlation between two cameras: (**a**) Camera 1 (camera under analysis) and detections in red color and (**b**) Camera 2 and detections in blue color. (**c**) Camera under analysis (Camera 1) with original red detections $D_{1}$ and transferred blue ones $D_{2}$. In this case, the optimal thresholds according to the correlation between both cameras are $0.4>\tau_{1}^{1,2}\leq 0.8$ and $0.2>\tau_{2}^{1,2}\leq 0.3$, respectively. All the images are cropped versions for visualization purposes.

Figure 3 shows one example of the correlation between two cameras: Camera 1 in Figure 3a and Camera 2 in Figure 3b. Figure 3c shows both camera detections ($D_{1}$ and $D_{2}$) in the camera under analysis and the final threshold configuration. Note how the correlation is able to avoid one false positive detection from each camera and to detect the occluded person in Camera 2, but not occluded in Camera 1.

**Figure 4:**
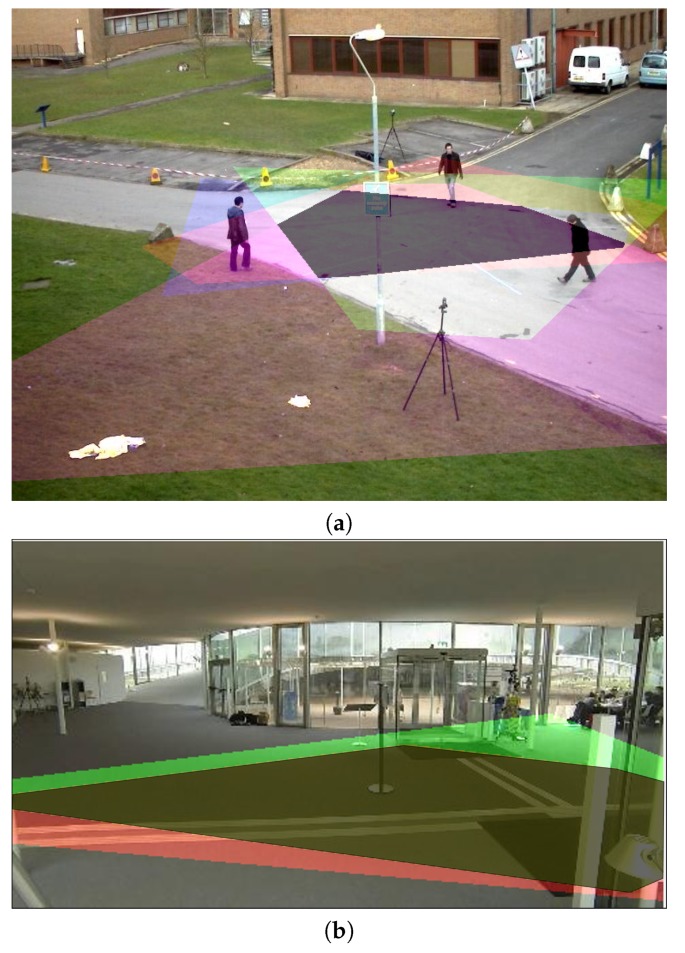
PETS2009 (**a**) and EPFL-RLC (**b**) view planes of each camera (five and three cameras, respectively) and the common field of view of all cameras.

The correlation is only coherent to be carried out in the common field of view of all cameras, since otherwise, disjoint sets would be correlated and the process would not be useful. To locate the common field of view, the ground plane of each camera is transferred to the desired point of view. Visual examples of this process are shown in Figure 4a, in which the plane of each camera is represented with a different color and the common field of view of all the cameras has been darkened to ease its localization. Since the common field of view is defined in the ground plane (ground floor plane in our scenario), the correlation and evaluation process will only take into account those pedestrians whose projected bounding box base is included in the common field of view (see Section 3 and Figure 2 for more details).

## 5. Experimental Results

### 5.1. Experimental Setup

The objective of this section is to evaluate the presented framework in order to validate the detection improvements. Two datasets, which contain overlapping multi-camera environments, have been considered for the evaluation. The PETS 2009 dataset (http://www.cvg.reading.ac.uk/PETS2009/a.html) presents outdoor sequences from a typical surveillance setup. We consider the available ground truth, which improves direct transfer cameras’ combination results of bounding boxes from [17] and sequences S2-L1and S3MF1, which are the only ones that contain available and synchronized frames for Cameras 1, 5, 6, 7, and 8 (five cameras in total renamed to cam1, cam2...,cam5). The EPFL-RLCdataset (https://cvlab.epfl.ch/data/rlc) was recorded at the EPFL Rolex Learning Center using three static HD cameras. The complete ground truth was not available, so we manually annotated the bounding boxes of the detections for the first 2000 frames of Camera 1 (cam1). We make this ground truth publicly available upon request. Both datasets were calibrated using the Tsai calibration [18], and the calibration files are included in the respective websites of the authors.

The detection performance was evaluated by Precision (P), Recall (R), and F-score (F) metrics for each frame. We considered the mean F-score for all sequence frames as the final performance value. Table 1 includes a short description of the experimental dataset, and Figure 4 includes the PETS2009 and EPFL-RLC view planes of each camera (five and three cameras, respectively) and the common field of view of all cameras. With respect to the detection algorithms, we consider four people detectors with publicly-available implementations: DPM [11] (Inria model), ACF [12], faster R-CNN [13] (VGG model), and YOLO9000 [14].

In order to evaluate the improvement of the framework, we compared the results with the absence of threshold adaptation, i.e., the use of an optimal threshold determined and fixed previously during training time. In our experiments, we learned offline this Optimal Fixed Threshold (OFT) with the training dataset VOC2012 (Visual Object Classes Challenge 2012 [19]).

### 5.2. Proposal Results

In order to evaluate the performance, we always transferred all the cameras to the reference point of view of cam1, since it was the one with the annotated ground truth. Table 2 shows the results obtained with the faster R-CNN detector and using each camera transferred detection (from cam1–cam3/5) in terms of Precision (P), Recall (R), and F-score (F). Table 3 shows the performance improvements in terms of F-score (F) versus the use of the Optimal Fixed Threshold (OFT). The results show clearly how cam1 presented always better performance (both OFT and the proposed approach) than the other cameras; as expected, since it was the one evaluated over its own viewpoint and therefore had a better view of the scene. Thanks to the proposed adaptation framework, the detection performance of every point of view was significantly improved even in the case of cam1. As expected, those with worse performance (from cam2–cam3/5) obtained higher improvements around 25%, whilst cam1, with better original performance, obtained between 2 and 13% improvement.

**Figure 5:**
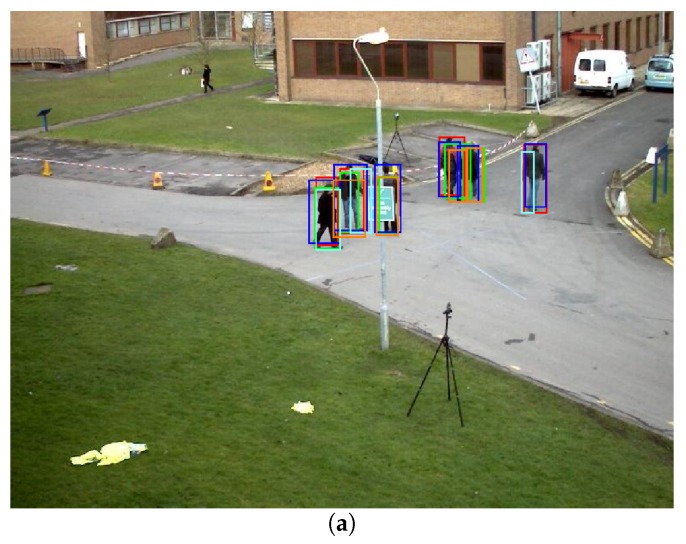

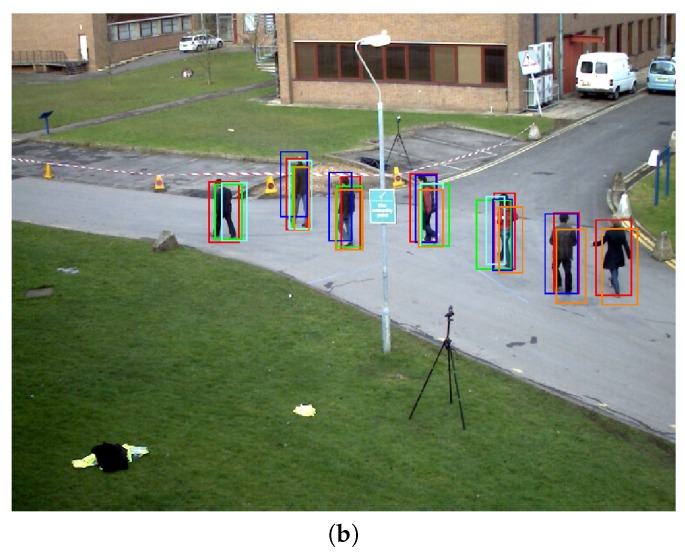
Visual examples of the faster R-CNN [13] detections of all cameras transferred to the evaluation viewpoint. Each camera bounding box is represented with a different color. Sequence PETS S2-L1, Frame 102 (**a**), and sequence PETS S3MF1, Frame 78 (**b**).

The results obtained after evaluating the four detectors on the sequences of the two datasets are presented in Table 4. The improvement obtained by applying the transfer of detections between cameras and the correlation framework was greater for detectors with worse performance (in this case, DPM, followed by ACF), because, as expected, the improvement margin was greater. For better performing detectors (faster R-CNN and YOLO9000), an improvement of the results was also achieved for all cases. Figure 5 shows two examples of the people detections (faster R-CNN) of all cameras transferred to the evaluation viewpoint, in which the bounding boxes of each camera are represented with a different color.

In addition to these improvements, the proposed approach avoided the critical task of selecting a static threshold fixed offline during training time for every possible scenario. We were able to choose the optimal threshold online automatically in every frame with a minimum requirement of one detector and two cameras.

According to the computational cost, each detector’s results had been obtained with the available code, implemented with different tools and programming languages, so a fair comparison was not possible. The computational cost of the detections was not treated in this paper, as this aspect was analyzed by the authors. The used DPM approach was implemented with MATLAB, and the computational cost was about 2 s per frame, considering an image of 352 × 288 pixels. The ACF detector was implemented in MATLAB, and the computational cost was about 20–30 ms per frame with 352 × 288 images. The used faster RCNN approach was implemented with MATLAB and Caffe, and the computational cost was about 150–200 ms per frame (faster RCNN, VGG-16 with GPU), considering an image of 500 × 375 pixels. The used YOLO9000 approach was implemented in C and CUDA (GPU), and the computational cost was about 25–35 ms per frame, considering an image of 500 × 375 pixels.

Our experiments have been performed on a Pentium i5 with a central processing unit with a frequency of 2.6 GHz and 8 GB random access memory. The proposed approach included two main tasks: the detection transfer and the correlation stage. Both stages were implemented in MATLAB. Table 5 includes computational cost results per frame in milliseconds (ms) of both the transfer and correlation stages of each video sequence. Note that the computational cost depended mainly on the number of detections and not the frame resolution. The computational cost of the detection transfer, once the homography transfer was initially estimated, was about 300–500 ms per frame. The correlation stage can be parallelizable by pairs of cameras, and the computational cost was about 5–10 ms per frame.

## 6. Conclusions and Future Work

We present a framework to choose the optimal people detector threshold automatically during runtime. The proposal accurately transfers detector results between camera viewpoints and then exploits the correlation among multiple camera detections transferred to a common camera to determine the best threshold for each camera. The proposed approach is capable of working over standard state-of-the-art detector outputs (bounding boxes), so any kind of detector and object model can be considered. The cylinder model may need to be adapted in other cases in which the object has a very unbalanced length-width aspect ratio (for example, a car or van). This framework allows the automatic threshold parametrization without requiring any model (re-)training process and, therefore, is completely online.

For future work, more object detectors can be considered. Other additional optimal parametrization can be considered and not only the detection threshold; for example, the position of the bounding box, the scale of detected objects, the pose, etc. Furthermore, following [15], multiple and different detectors could be also applied for each camera and combined simultaneously, in order to further improve the results.

## Figures and Tables

**Table 1:** Description of the experimental dataset.

Sequence	# of Cameras	# of Frames	# of Pedestrians per Frame
PETS S2-L1	5	795	1.8
PETS S3MF1	5	107	1.9
EPFL-RLC	3	2000	6.1

**Table 2:** Precision (P), Recall (R), and F-score (F) values obtained for each Camera (Cam) transferred detection with the faster R-CNN detector [13].

	Cam1			Cam2			Cam3			Cam4			Cam5		
Sequence	P	R	F	P	R	F	P	R	F	P	R	F	P	R	F
PETS S2-L1	**0.78**	**0.74**	**0.74**	0.63	0.56	0.58	0.56	0.55	0.54	0.56	0.55	0.53	0.56	0.53	0.53
PETS S3MF1	**0.68**	**0.67**	**0.66**	0.60	0.53	0.55	0.50	0.50	0.49	0.51	0.47	0.48	0.48	0.44	0.46
EPFL-RLC	**0.91**	**0.66**	**0.73**	0.80	0.56	0.63	0.71	0.45	0.52						

**Table 3:** F-score (F) results’ values obtained for each camera transferred detection with the faster R-CNN detector [13] and comparison with the use of the Optimal Fixed Threshold (OFT).

	Cam1			Cam2			Cam3			Cam4			Cam5		
Sequence	OFT	Ours	%Δ	OFT	Ours	%Δ	OFT	Ours	%Δ	OFT	Ours	%Δ	OFT	Ours	%Δ
PETS S2-L1	0.69	**0.74**	7.6	0.47	0.58	23.6	0.43	0.54	25.2	0.43	0.53	25.6	0.43	0.53	24.2
PETS S3MF1	0.65	**0.66**	2.6	0.45	0.55	23.9	0.39	0.49	26.2	0.38	0.48	26.5	0.36	0.46	28.1
EPFL-RLC	0.65	**0.73**	12.9	0.53	0.63	20.3	0.38	0.52	36.8						

**Table 4:** Precision (P), Recall (R), and F-score (F) values obtained for the four detection algorithms and comparison with the use of the Optimal Fixed Threshold (OFT).

	**DPM [11]**								
	**OFT**			**Ours**			%Δ		
Sequence	P	R	F	P	R	F	P	R	F
PETS S2-L1	0.41	0.33	0.35	0.54	0.41	0.45	**31.6**	**25.9**	**29.0**
PETS S3MF1	0.47	0.34	0.38	0.60	0.46	0.50	**25.8**	**35.9**	**31.8**
EPFL-RLC	0.70	0.22	0.32	0.80	0.44	0.49	**13.7**	**98.9**	**54.6**
	**ACF [12]**								
	**OFT**			**Ours**			%Δ		
Sequence	P	R	F	P	R	F	P	R	F
PETS S2-L1	0.76	0.66	0.69	0.81	0.71	0.74	**6.6**	**6.3**	**6.5**
PETS S3MF1	0.61	0.43	0.49	0.68	0.58	0.61	**12.6**	**33.9**	**25.6**
EPFL-RLC	0.80	0.36	0.45	0.85	0.46	0.54	**6.8**	**27.5**	**20.4**
	**Faster R-CNN [13]**								
	**OFT**			**Ours**			%Δ		
Sequence	P	R	F	P	R	F	P	R	F
PETS S2-L1	0.70	0.66	0.69	0.78	0.74	0.74	**11.3**	**11.5**	**7.6**
PETS S3MF1	0.65	0.63	0.65	0.68	0.67	0.66	**4.1**	**5.6**	**2.6**
EPFL-RLC	0.83	0.55	0.65	0.91	0.66	0.73	**9.5**	**20.2**	**12.9**
	**YOLO9000 [14]**								
	**OFT**			**Ours**			%Δ		
Sequence	P	R	F	P	R	F	P	R	F
PETS S2-L1	0.74	0.65	0.68	0.81	0.71	0.74	**9.2**	**10.5**	**9.9**
PETS S3MF1	0.64	0.58	0.60	0.69	0.63	0.65	**7.8**	**9.0**	**8.7**
EPFL-RLC	0.81	0.59	0.67	0.92	0.60	0.74	**13.4**	**1.9**	**11.2**

**Table 5:** Computational cost results obtained with the faster R-CNN detections [13] per frame in milliseconds (ms) of both the transfer and correlation stages of each video sequence.

Sequence	# of Pedestrians Per Frame	Detection Transfer Stage (ms)	Correlation Stage (ms)	Total (ms)
PETS S2-L1	1.8	387.2	4.3	391.5
PETS S3MF1	1.9	294.8	5.8	300.6
EPFL-RLC	6.1	554.1	10.1	564.2
